# Targeted Next-Generation Sequencing Identified Novel Compound Heterozygous Variants in the *PTPRQ* Gene Causing Autosomal Recessive Hearing Loss in a Chinese Family

**DOI:** 10.3389/fgene.2022.884522

**Published:** 2022-07-08

**Authors:** Yuan Jin, Xiao-Zhou Liu, Le Xie, Wen Xie, Sen Chen, Yu Sun

**Affiliations:** ^1^ Department of Otorhinolaryngology, Union Hospital, Tongji Medical College, Huazhong University of Science and Technology, Wuhan, China; ^2^ Tongji Medical College, Institute of Otorhinolaryngology, Huazhong University of Science and Technology, Wuhan, China

**Keywords:** *PTPRQ* gene, novel compound heterozygous mutation, targeted next-generation sequencing, hearing loss, autosomal recessive inheritance

## Abstract

Hearing loss is among the most common congenital sensory impairments. Genetic causes account for more than 50% of the cases of congenital hearing loss. The *PTPRQ* gene, encoding protein tyrosine phosphatase receptor Q, plays an important role in maintaining the stereocilia structure and function of hair cells. Mutations in the *PTPRQ* gene have been reported to cause hereditary sensorineural hearing loss. By using next-generation sequencing and Sanger sequencing, we identified a novel compound heterozygous mutation (c.997 G > A and c.6603-3 T > G) of the *PTPRQ* gene in a Chinese consanguineous family. This is the first report linking these two mutations to recessive hereditary sensorineural hearing loss. These findings contribute to the understanding of the relationship between genotype and hearing phenotype of *PTPRQ*-related hearing loss, which may be helpful to clinical management and genetic counseling.

## Introduction

Hearing loss is the most common sensorineural disorder affecting approximately 6.1% of the world population (Qian et al., 2020; Zhang et al., 2021a; Fu et al., 2021; Lv et al., 2021). It is estimated that more than half of the hearing loss cases are attributable to genetic factors (He et al., 2017; Wang et al., 2022), while the other half of hearing loss cases could be caused by ototoxic drugs, such as aminoglycosides and anti-tumor drugs, aging, excessive noise exposure, and infections (Li et al., 2018a; Li et al., 2018b; Liu et al., 2019a; Cheng et al., 2019; Han et al., 2020; He et al., 2020; Zhong et al., 2020; Zhou et al., 2020; Liu et al., 2021a; Zhang et al., 2021b; Guo et al., 2021; He et al., 2021; Bu et al., 2022; Fu et al., 2022; Jiang et al., 2022). The functions of these hearing loss genes play an essential role in the development and function of hair cells and synaptic transmission of spiral ganglion neurons (Wang et al., 2017; Zhu et al., 2018; Cheng et al., 2021). Thus, hearing loss is often induced by the loss of sensory hair cells and spiral ganglion neurons (Liu et al., 2019b; Guo et al., 2019; He et al., 2019; Qi et al., 2019; Chen et al., 2021; Hu et al., 2021; Wei et al., 2021; Guo et al., 2022; Hu et al., 2022; Jiang et al., 2022) in the inner ear cochlea. Up to 30/8/2021, at least 124 genes have been identified associated with non-syndromic hearing loss genes (https://hereditaryhearingloss.org/).

The *PTPRQ* gene, located in the DFNB84 region of chromosome 12q21.31, is comprised of 58 exons (Schraders et al., 2010). The transcript levels of *PTPRQ* are the highest in fetal kidneys, followed by fetal lungs and fetal cochlea (Schraders et al., 2010). In the cochlea, the PTPRQ protein (Protein Tyrosine Phosphatase Receptor Type Q, which encodes 2,299 amino acids) expresses in the basal region of the stereocilia of hair cells (Ozieblo et al., 2019). Particularly, the PTPRQ protein has a higher expression level in the basal turn of the cochlea corresponding to high-frequency hearing (Goodyear et al., 2003). Studies have shown that PTPRQ is indispensable for the formation of hair bundles. In the early postnatal *Ptprq* −/− mouse model, elongated and fused stereocilia in inner hair cells (IHCs), shortened stereocilia in outer hair cells (OHCs), and loss of hair bundles in both OHCs and IHCs were observed (Goodyear et al., 2003). In the adult *Ptprq* −/− mice, almost all hair cells had degenerated and even the organ of Corti was missing. In addition, PTPRQ protein forms a complex with myosin VI to tether the membrane of the stereocilia to stereocilia, causing reorganization of the actin cytoskeleton, and plays an important role in the mechanical transduction and adaptation of hair cells (Takenawa and Itoh, 2001; Hirono et al., 2004; Sakaguchi et al., 2008). Studies in families with *PTPRQ* mutations show that mutants of the *PTPRQ* gene could cause autosomal recessive or autosomal dominant congenital sensorineural hearing loss, damage all frequency or high frequency, with or without vestibular dysfunction in infancy or early childhood (Li et al., 2018a; Li et al., 2018b; Liu et al., 2019a; Cheng et al., 2019; Zhou et al., 2020; Liu et al., 2021a; Guo et al., 2021; He et al., 2021; Bu et al., 2022; Fu et al., 2022; Jiang et al., 2022). The hearing loss was progressive in some cases. In addition, transcription of *PTPRQ* was highly expressed in adult lung and heart tissues, and there has been no significant evidence showing dysfunction of organs except that of the cochlea (Schraders et al., 2010).

Until now, cases of *PTPRQ*-related hearing loss rarely have been reported (summarized in Tables 1, 2). More cases of gene mutation need to be collected to understand the molecular mechanism. Here, we report a novel heterozygous *PTPRQ* mutation in a Chinese family, which might be helpful to establish a better understanding of the relationship between *PTPRQ* and the phenotype.

**TABLE 1:** Standard and Colloquial nomenclature for *PTPRQ* mutations and variants.

DNA sequence change*	Amino acid change	Commonly used colloquial nomenclature	Site of mutation	Type of mutation
c.4006C > T	p.Gln1336X	Q1336X	Exon 24	Nonsense
c.6881G > A	p.Trp2294X	W2294X	Exon 45	Nonsense
c.1973T > C	p.Val658Ala	V658A	Exon 14	Missense
c.4472C > T	p.Thr1491Met	T1491M	Exon 26	Missense
c.5592dup	p.(Glu134Glyfs*6)	—	Exon 32	Frame shift
c.6080dup	p.(Asn2027Lys*9)	—	Exon 38	Frame shift
c.6881G > A	p.Trp2294X	T2294X	Exon 45	Nonsense
c.16_17insT	p.Leu8fsX18	128insT	Exon 1	Frame shift
c.2714delA	p.Glu909fsX922	2825delA	Exon 18	Frame shift
c.55-2A > G	—	c.166-2A > G	Intron 1	Splice site
c.2599T > C	p.Ser867Pro	S867P	Exon 17	Missense
c.3125A > G	p.Asp1042Gly	D1042G	Exon 20	Missense
c.5981A > G	p.Glu1994Gly	E1994G	Exon 37	Missense
c.1491T > A	p.Tyr497X (currently Tyr279X)	Y497X	Exon 10	Nonsense
c.1369A > G	p.Arg457Gly (currently Arg239Gly)	R457G	Exon 10	Missense
c.1285C > T	p.Gln429X	Q429X	Exon 9	Nonsense
c.1261C > T	p.Arg421X	R421X	Exon 9	Nonsense
c.166C > G	p.Pro56Ala	P56A	Exon 3	Missense
c.6453 + 3delA	—	c.6564 + 3delA	Intron 41	Splice site
c.4640T > C	p.Met1349Thr	M1349T	Exon 27	Missense
c.1057delC	p.Leu353SfsX8	1168delC	Exon 8	Frame shift

Nucleotide numbering is based on DNA reference sequence NM_001145026.2. The version number of this reference sequence may be frequently updated. The table was made with reference to previous literature (Shuji et al., 2007).

**TABLE 2:** *PTPRQ* mutations with hearing phenotypes in families.

Genotype	Protein domain	Inheritance pattern	Frequencies of hearing loss	Vestibular dysfunction	Phenotype	Reference
c.4006C > T/c.4006C > T	FN III	Autosomal recessive	Not mentioned	Yes	Hearing loss	Paridhy et al. (2021)
c.6881G > A/WT	—	Autosomal dominant	Mid to high frequencies	No	Mild to severe hearing loss	Eisenberger et al. (2018)
c.1973T > C/c.4472C > T	FN III	Autosomal recessive	All frequencies	No	Severe to profound hearing loss	Lv et al. (2021)
c.5592dup/c.5592dup	FN III	Autosomal recessive	Not mentioned	No	Profound hearing loss	Ammar-Khodja et al. (2015)
c.6080dup/c.6080dup	FN III	Autosomal recessive	Not mentioned	No	Profound hearing loss	Ammar-Khodja et al. (2015)
c.6881G > A/WT	—	Autosomal dominant	Mid to high frequencies	No	Severe hearing loss	Ozieblo et al. (2019)
c.16_17insT/c.2714delA	—/FN III	Autosomal recessive	All frequencies	No	Severe hearing loss	Sang et al. (2015)
c.55-2A > G/c.55-2A > G	FN III	Autosomal recessive	Mid to high frequencies	No	Severe to profound hearing loss	Mahmood et al. (2021)
c.2599T > C/c.2599T > C	FN III	Autosomal recessive	Not mentioned	Not mentioned	Hearing loss	Talebi et al. (2018)
c.3125A > G/c.5981A > G	FN III/—	Autosomal recessive	All frequencies	No	Moderate to profound hearing loss	Gao et al. (2015)
c.1491T > A/c.1491T > A	—	Autosomal recessive	All frequencies	Yes	Profound hearing loss	Schraders et al. (2010)
c.1369A > G/c.1369A > G	—	Autosomal recessive	All frequencies	Yes	Moderate hearing loss	Schraders et al. (2010)
c.1285C/T/c.1285C/T	FN III	Autosomal recessive	All frequencies	Not mentioned	Moderate to severe hearing loss	Shahin et al. (2010)
c.1261C > T/c.1261C > T	FN III	Autosomal recessive	Mid to high frequencies	Yes	Profound hearing loss	Sakuma et al. (2015)
c.166C > G/c.1261C > T	FN III	Autosomal recessive	All frequencies	No	Profound hearing loss	Sakuma et al. (2015)
c.6453 + 3delA/c.4640T > C	—/FN III	Autosomal recessive	All frequencies	No	Moderate hearing loss	Sakuma et al. (2015)
c.1057delC/c.1057delC	FN III	Autosomal recessive	Not mentioned	Not mentioned	Hearing loss	Yang et al. (2021)

Hearing loss was classified as mild (20–40 dB), moderate (41–60 dB), severe (61–90 dB), or profound (>90 dB); low frequencies mean 125–500 Hz; medium frequencies mean 500–2000 Hz; high frequencies mean 2000–8000 Hz; FN III, fibronectin type III protein domain.

## Materials and Methods

### Family Description

**FIGURE 1:**
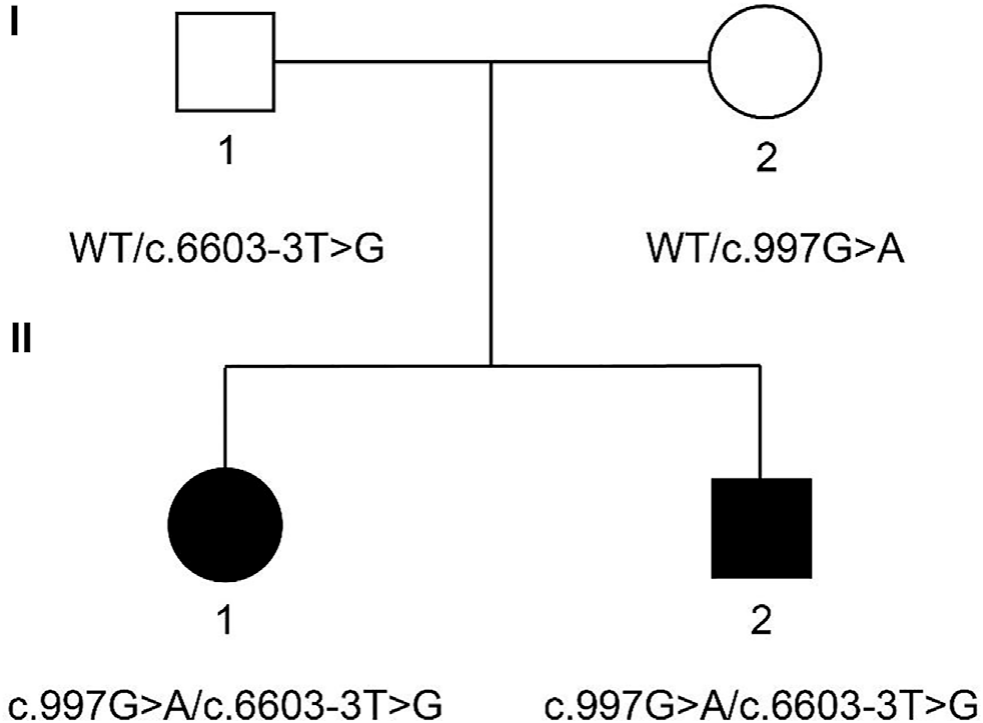
Family pedigree of two probands. Probands II-1 and II-2 carry compound heterozygous mutation c.997 G > A (chr12:80862555) and c.6603-3 T > G (chr12:81066945) of PTPRQ. The mother of the probands carries heterozygous mutation c.997 G > A. The father of the probands carries heterozygous mutation c.6603-3 T > G. Probands are marked in black. WT, wild type.

The family members are Han Chinese. Proband II-1 is a 29-year-old female. Proband II-2 is a 23-year-old male. Both probands had failed to pass the hearing screening and were diagnosed with sensorineural hearing loss. Neither parent of two probands exhibited similar hearing loss or vestibular dysfunction (Figure 1).

### Clinical Examination

Both probands underwent audiological examination such as the otoscopic examination, auditory immittance, and auditory steady-state–evoked responses (ASSR). Computed tomography (CT) and magnetic resonance imaging (MRI) of the temporal bone showed no abnormal malformations. Parents reported no history of miscarriage or stillbirth. The physical examination, otoscopy, and medical history were performed at the outpatient clinic of Wuhan Union Medical College Hospital.

### Mutation Detection and Analysis

The method has been described in detail in our previous articles (Chen et al., 2020; Liu et al., 2021b). Briefly, the two probands and their parents each contributed 3–5 ml of venous peripheral blood after the participants had given their informed consent. Genomic DNA was isolated from the blood samples using the QIAamp DNA Blood Midi Kit (Qiagen Inc., Hilden, Germany). Fragmentation of the genomic DNA was performed by using a Covaris LE220 ultrasonicator (Covaris Inc., Woburn, Massachusetts, United States) to generate a paired-end library. The library was enriched after hybridization, elution, and post-capture amplification. The amplified DNA library was sequenced on the BGISEQ-500 platform. Sequencing data were compared with the human genome reference (GRCh37/hg19) to detect target regions, single-nucleotide variants (SNVs), and INDEL calling. Identified SNVs and indels were compared with the information available in multiple databases, such as the National Center for Biotechnology Information GenBank database (https://www.ncbi.nlm.nih.gov/nuccore/), the Database of Single Nucleotide Polymorphisms (dbSNP) (http://www.ncbi.nlm.nih.gov/projects/SNP/), and the 1000 Genomes Database (https://www.internationalgenome.org). According to the sequencing results of two probands, Sanger sequencing was performed to confirm whether their parents had the same mutations. Using online tools such as HSF (http://www.umd.be/HSF3/HSF.shtml), FF (http://www.fruitfly.org/seq_tools/splice.html), SpliceAI (https://spliceailookup.broadinstitute.org/), an assessment was made to determine whether mutations occurring in the introns affected hnRNA splicing.

## Results

### Clinical Data

**FIGURE 2:**
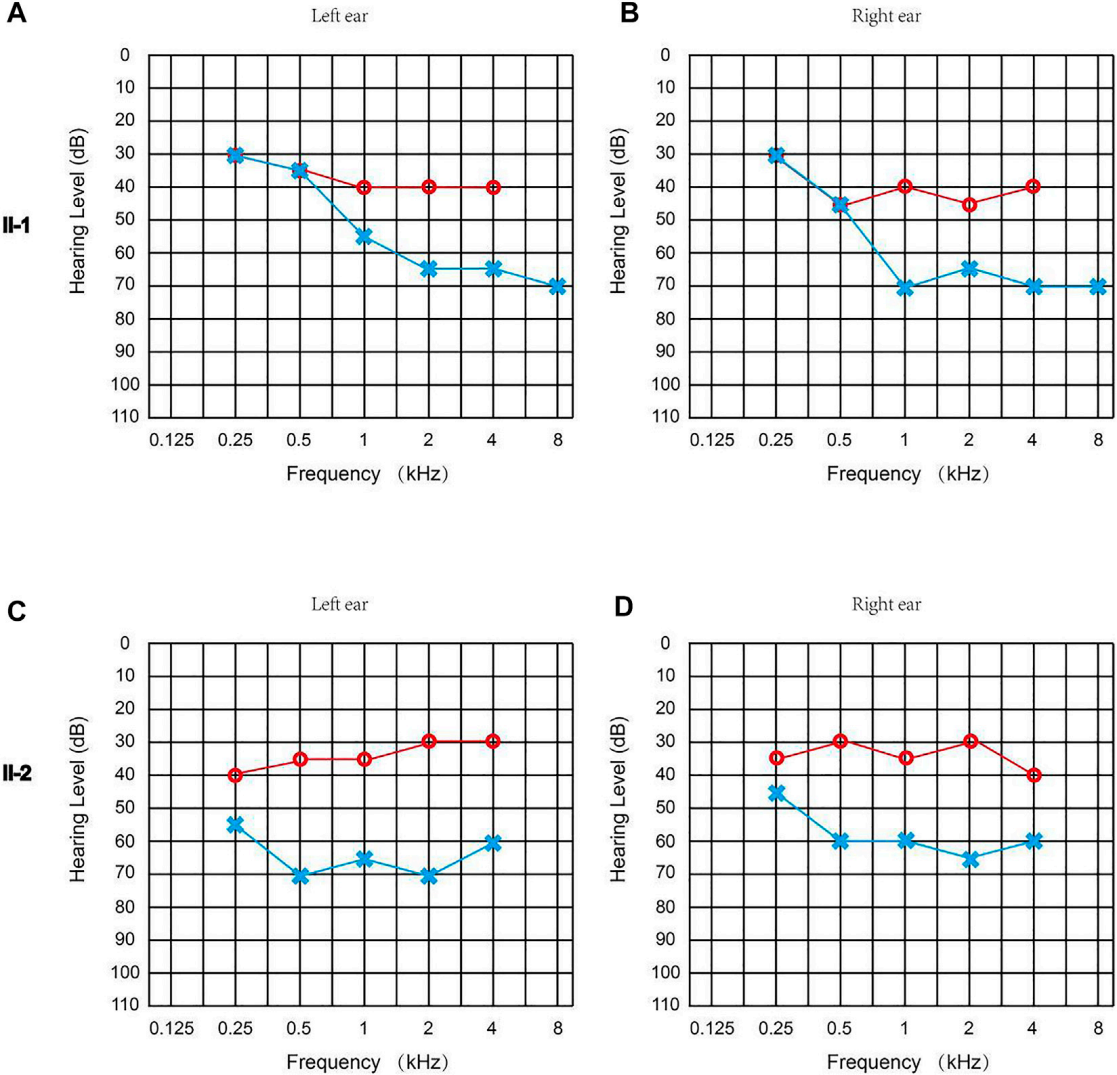
Clinical audiology examination of the probands. **(A)** ASSR of Proband II-1 (left ear): 30, 35, 55, 65, 65, and 70 dBnHL at 0.25, 0.5, 1, 2, 4, and 8 kHz. **(B)** ASSR of Proband II-1 (right ear): 30, 45, 70, 65, 70, and 70 dBnHL at 0.25, 0.5, 1, 2, 4, and 8 kHz. **(C)** ASSR of Proband II-2 (left ear): 55, 70, 65, 70, and 60 dBnHL at 0.25, 0.5, 1, 2, and 4 kHz. **(D)** ASSR of the Proband II-2 (right ear): 45, 60, 60, 65, and 60 dBnHL at 0.25, 0.5, 1, 2, and 4 kHz. The hearing threshold of two probands with a hearing aid is marked in red, and the hearing threshold of two probands without a hearing aid is marked in blue.

Both patients failed the newborn hearing screening and were diagnosed with congenital sensorineural hearing loss. The ASSR of Proband II-1 showed the thresholds of the left ear were 30, 35, 55, 65, 65, and 70 dBnHL at 0.25, 0.5, 1, 2, 4, and 8 kHz, while the thresholds of the right ear were 30, 45, 70, 65, 70, and 70 dBnHL at 0.25, 0.5, 1, 2, 4, and 8 kHz (Figures 2A,B). The ASSR of Proband II-2 showed that the thresholds of the left ear were 55, 70, 65, 70, and 60 dBnHL at 0.25, 0.5, 1, 2, and 4 kHz, while the thresholds of the right ear were 45, 60, 60, 65, and 60 dBnHL at 0.25, 0.5, 1, 2, and 4 kHz (Figures 2C,D). The temporal bone CT scan suggested that the shape and size of the bilateral cochleae were not obviously abnormal. Neither proband reported ever suffering from balance manifestation, tinnitus, or vertigo. In addition, the parents of the probands stated that both probands had no symptoms of falling down or frequent standing instability during their childhood. Physical examinations of the two probands revealed no signs of systemic illness. After wearing a hearing aid, the ASSR of Proband II-1 showed the thresholds of the left ear were 30, 35, 40, 40, and 40 dBnHL at 0.25, 0.5, 1, 2, and 4 kHz, while the thresholds of the right ear were 30, 45, 40, 45, and 40 dBnHL at 0.25, 0.5, 1, 2, and 4 kHz (Figures 2A,B); the ASSR of Proband II-2 showed the thresholds of the left ear were 40, 35, 35, 30, and 30 dBnHL at 0.25, 0.5, 1, 2, and 4 kHz, while the thresholds of the right ear were 35, 30, 35, 30, and 40 dBnHL at 0.25, 0.5, 1, 2, and 4 kHz (Figures 2C,D). Their parents had no history of hearing impairment, nor did their medical history include other organ disorders.

### Mutation Identification Data

**FIGURE 3:**
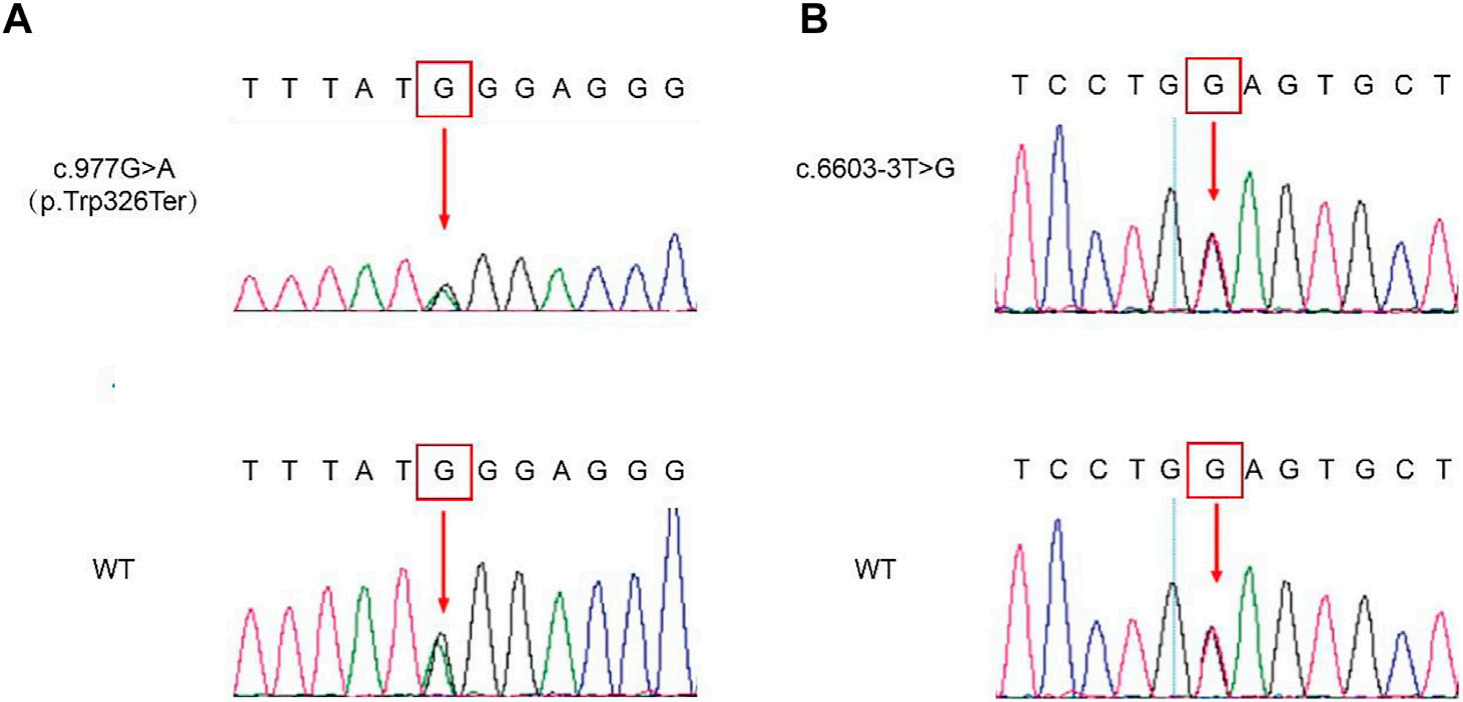
Genetic sequencing results of the probands and their parents. Mutated sequences of the identified c.997 G > A **(A)** and c.6603-3 T > G **(B)** variant. The red arrow indicates the site of the base deletion or substitution.

The genomic DNA sequences of the probands were compared with the human genome reference sequence (GRCh37/hg19). Both probands carried compound heterozygous mutations of *PTPRQ*: c.997 G > A and c.6603-3 T > G. The mutation c.997 G > A occurred in EX7/CDS7 in the *PTPRQ* gene, causing the substitution of no. 997 nucleotide from guanine to adenine (Figure 3A). The mutation c.6603-3 T > G is a splice mutation in Intron 42, causing the substitution of no. 6603-3 nucleotide from thymine to guanine (Figure 3B). The proband’s father and mother were heterozygous carriers of the c.997 G > A and c.6603-3 T > G mutations, respectively (Figure 1). The mutation c.6603-3 T > G of *PTPRQ* was inherited from the father and reported a minor allele frequency of 0.000078 in the gnomAD database. The allele frequency of this mutation in the East Asian population is 0.002118. The mutation c.997 G > A of *PTPRQ* was inherited from the mother, and no information on this mutation was found in the gnomAD database.

### Functional Analysis of the Mutant Protein

**FIGURE 4:**
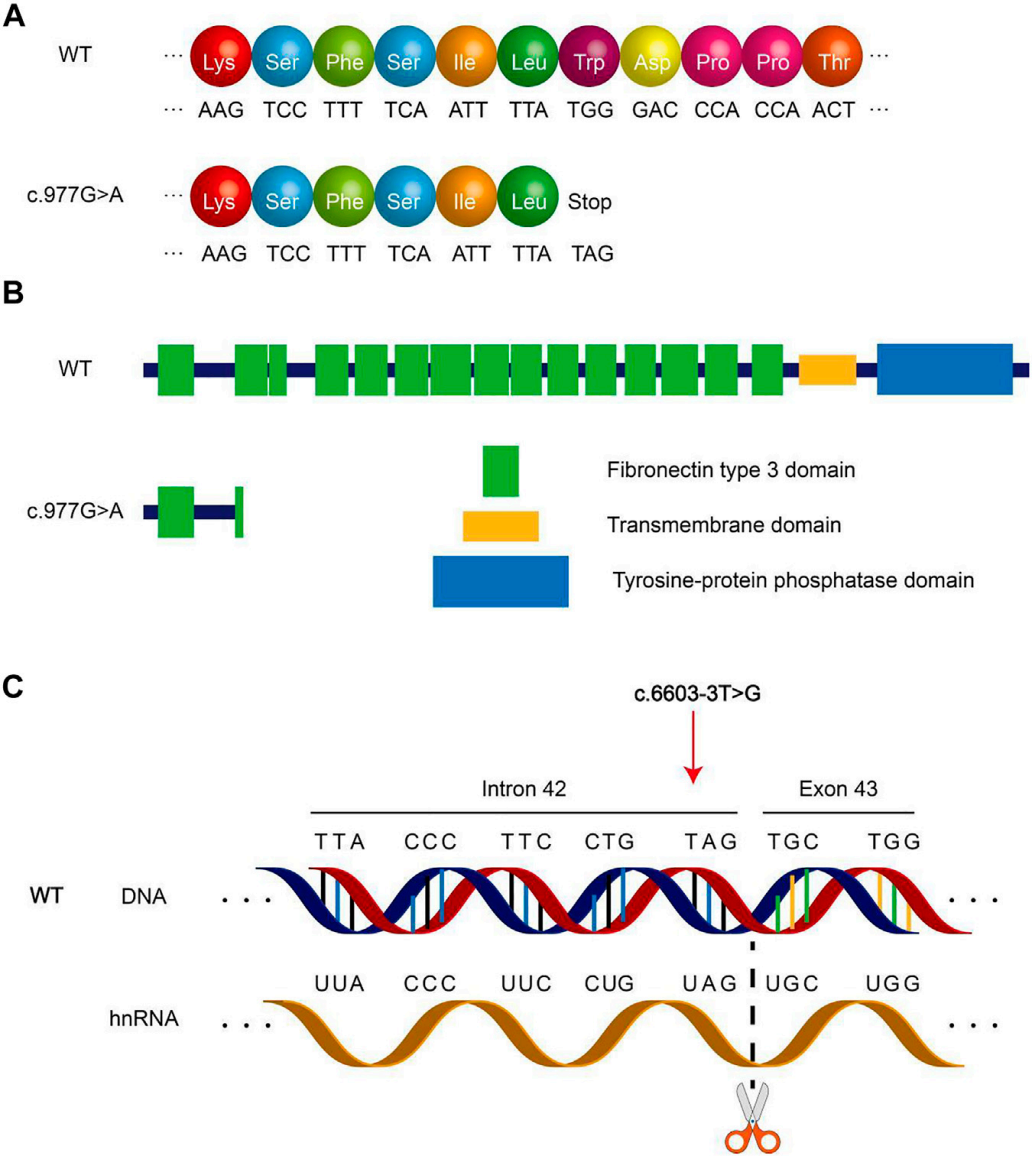
Schematic diagram of *PTPRQ* mutation. **(A)** Amino acid coding diagram of mutation c.997 G > A. **(B)** Schematic diagram of *PTPRQ* peptide chain truncation caused by mutation c.997 G > A. **(C)** Schematic diagram of mutation c.6603-3 T > G altering hnRNA splicing.

The PTPRQ protein is composed of three types of domains, namely, 18 fibronectin III repeats domain (FN III domain), transmembrane domain, and tyrosine–protein phosphatase domain (PTPase domain). The mutation c.997 G > A causes the original TGG at nucleotide nos. 976–978 to become TAG, which corresponds to the termination codon (Figure 4A). The mutation c.997 G > A occurs in the second fibronectin III domain. The mutation c.997 G > A resulted in a truncated protein of only 325 proteins. Thus, the following 1,974 amino acids after this site cannot be synthesized (Figures 4A,B). The mutation c.6603-3 T > G causes the original thymine at nucleotide no. 6603-3 in Intron 42 to become guanine, which is near the splice site between Intron 42 and Exon 43 (Figure 4C). This mutation type often results in abnormal splicing of hnRNA. The amino acid encoded by Exon 43 is involved in the formation of the PTPase domain. We assessed whether mutation c.6603-3 T > G affects *PTPRQ* hnRNA splicing using the online bioinformatics database. All three database tools, HSF(http://www.umd.be/HSF3/HSF.shtml), FF(http://www.fruitfly.org/seq_tools/splice.html), and SpliceAI (https://spliceailookup.broadinstitute.org/), suggest that mutations c.6603-3 T > G will change the original acceptor site and hnRNA splicing. However, patients did not intend to participate in the minigene splicing assay. Therefore, we did not verify the effect of mutation c.6603-3 T > G in *in vitro* experiments.

## Discussion

Both probands failed the newborn hearing screening and were diagnosed with congenital sensorineural hearing loss. The proband’s parents were consanguineous. However, they did not provide more detailed information on family members. By using an approach of next-generation sequencing and the Sanger sequencing method, we identified c.997 G > A and c.6603-3 T > G of *PTPRQ* in the family, as the probable cause of sensorineural hearing loss.

Mutation c.997 G > A of *PTPRQ* causes termination of protein synthesis. The mutant protein contains only one complete fibronectin III domain, the other domains are completely deleted (Figure 4B). Such proteins basically lose their function. Usually, the 3′ end splicing site of an intron ends with an AG. Mutations occurring in introns and close to this region may result in changes in the transcription sequence, affecting the nucleotide sequence of the final transcript. The mutation c.6603-3 T > G of *PTPRQ* occurs in Intron 42, a region near the boundary of Intron 42 and Exon 43, which may lead to changes in hnRNA splicing. Our assessment of this mutation using database tools revealed that the mutation resulted in the disappearance of the original splice site between Intron 42 and Exon 43, possibly resulting in the skipping of Exon 43 during transcription. More seriously, the mutation c.6603-3 T > G of *PTPRQ* may also lead to all subsequent changes in the amino acid sequence and protein domains. Exon 43 correlates with the synthesis of PTPase domains of PTPRQ proteins. According to the assessment (by HSF, FF, and SpliceAI), mutation c.6603-3 T > G of *PTPRQ* may cause altered hnRNA splicing, which can lead to changes in the composition and function of the PTPase domains. Neither mutant protein in two probands lacks the PTPase domains that are critical for the normal functioning of the PTPRQ protein. The PTPase domains have phosphatidylinositol phosphatase activity, so the PTPRQ protein can regulate the local phosphoinositides' concentration in a specific area. Furthermore, phosphoinositides play a role in cell growth, polarity, and movement by regulating the reorganization of actin filaments (Sakaguchi et al., 2008). In the inner ear, *PTPRQ* maintains the stability of the stereocilia bundle on hair cells (Goodyear et al., 2012). Deletion of the PTPase domain may lead to the degradation of stereocilia bundles. The final mutated protein results in impaired mechanotransduction function of hair cells, resulting in hearing loss.

A total of 21 *PTPRQ* mutations have been reported before this article (Table 1). The nucleotide and amino acid changes for these mutations are summarized in Table 1. Two mutations mentioned in this article, namely, mutations c.997 G > A of *PTPRQ* and c.6603-3 T > G of *PTPRQ*, have not been reported before. According to reports, some patients with *PTPRQ* mutations suffer from mild- to high-frequency hearing loss, while others suffer hearing loss in all frequencies (Han et al., 2020; He et al., 2020; Zhang et al., 2021a; Zhang et al., 2021b; Lv et al., 2021). The relationship between the genotype and hearing phenotype in deaf patients carrying PTPRQ mutations is summarized in Table 2. In the present study, Proband II-1 suffered from mild- to high-frequency hearing loss, while Proband II-2 with the same mutations suffered hearing loss in all frequencies. Although the hearing loss may progress steadily, the low-frequency hearing threshold of Proband II-2 is much higher than Proband II-1. In addition, some patients with *PTPRQ* mutations have been reported to sustain vestibular dysfunction such as tinnitus or vertigo (Cheng et al., 2019; Zhang et al., 2021b; He et al., 2021; Fu et al., 2022), while others and the probands in this study did not show the same symptoms. We emphasize again that there may be no obvious correlation between genotype and phenotype of *PTPRQ*.

No obvious disease of other organs was found in participants until now. However, this risk remains due to the limited number of reported cases of *PTPRQ* mutations. We recommend cochlear, kidney, lung, and thyroid function tests for patients with *PTPRQ* mutations. In addition, we noticed that the c.6603-3 T > G mutation of *PTPRQ* was only found in the East Asian population in the GnomAD database. In the future, we will continue to pay attention to *PTPRQ*-related reports carried out in the East Asian population.

## Conclusion

We identified a novel compound heterozygous mutation of *PTPRQ* (c.997 G > A and c.6603-3 T > G) in a Chinese family with non-syndromic sensorineural hearing loss. Our study expanded the spectrum of *PTPRQ* mutations. These findings contribute to the understanding of the relationship between genotype and hearing phenotype of *PTPRQ*-related hearing loss, which may be helpful to clinical management and genetic counseling.

## Data Availability

The datasets for this article are not publicly available due to concerns regarding participant/patient anonymity. Requests to access the datasets should be directed to the corresponding author.
